# Differential expression of microRNAs and other small RNAs in muscle tissue of patients with ALS and healthy age-matched controls

**DOI:** 10.1038/s41598-018-23139-2

**Published:** 2018-04-04

**Authors:** Anja Kovanda, Lea Leonardis, Janez Zidar, Blaž Koritnik, Leja Dolenc-Groselj, Stanislava Ristic Kovacic, Tomaž Curk, Boris Rogelj

**Affiliations:** 1 Department of Biotechnology, Jozef Stefan Institute, Jamova 39, Ljubljana, Slovenia; 2 Institute of Clinical Neurophysiology, Division of Neurology, University Medical Centre Ljubljana, Zaloška cesta 7, Ljubljana, Slovenia; 3 Department of Neurology, Faculty of Medicine, University of Ljubljana, Korytkova ulica 2, 1000 Ljubljana, Slovenia; 4 University of Ljubljana, Faculty of Computer and Information Science, Večna pot 113, 1000 Ljubljana, Slovenia; 5 Biomedical Research Institute BRIS, Ljubljana, 1000 Slovenia; 6 Faculty of Chemistry and Chemical Technology, University of Ljubljana, Večna pot 113, Ljubljana, 1000 Slovenia

## Abstract

Amyotrophic lateral sclerosis is a late-onset disorder primarily affecting motor neurons and leading to progressive and lethal skeletal muscle atrophy. Small RNAs, including microRNAs (miRNAs), can serve as important regulators of gene expression and can act both globally and in a tissue-/cell-type-specific manner. In muscle, miRNAs called myomiRs govern important processes and are deregulated in various disorders. Several myomiRs have shown promise for therapeutic use in cellular and animal models of ALS; however, the exact miRNA species differentially expressed in muscle tissue of ALS patients remain unknown. Following small RNA-Seq, we compared the expression of small RNAs in muscle tissue of ALS patients and healthy age-matched controls. The identified snoRNAs, mtRNAs and other small RNAs provide possible molecular links between insulin signaling and ALS. Furthermore, the identified miRNAs are predicted to target proteins that are involved in both normal processes and various muscle disorders and indicate muscle tissue is undergoing active reinnervation/compensatory attempts thus providing targets for further research and therapy development in ALS.

## Introduction

Amyotrophic lateral sclerosis (ALS) is a late-onset disorder primarily affecting upper and lower motor neurons leading to progressive and severe skeletal muscle atrophy. Whether the denervation is initiated primarily in the CNS or by the muscle itself remains under debate^1^. What is becoming increasingly clear, however, is that a complex molecular interplay contributes to this disorder, of which many components are closely involved in RNA metabolism.

One of the main pathogenic features of ALS are cytoplasmic aggregates of an otherwise predominantly nuclear DNA- and RNA-binding protein TDP-43 (TAR DNA-binding protein) in affected neurons, however, mutations of this protein in patients are too rare to explain this phenomenon^2–4^. TDP-43 is an RNA processing protein and is known to be intricately involved in RNA metabolism^5–7^. In addition to TDP-43, mutations and mislocalizations of other RNA-binding proteins, such as FUS and other hnRNPs (heterogeneous ribonucleoprotein) have also been shown to be associated with ALS^8–13^. The intronic (G_4_C_2_) hexanucleotide repeat expansion within the C9ORF72 gene has been shown to be the main genetic feature of ALS^14–16^. As well as giving rise to exotic DNA features such as G-quadruplexes and i-motifs^17,18^, the expanded repeats undergo both aberrant and unconventional processing (reviewed in Vatovec *et al*.^19^), which further supports disease-associated changes in RNA metabolism as a core mechanism in ALS pathogenesis.

MicroRNAs (miRNAs) are around 22 nucleotide long non-coding RNA molecules that serve as regulators of transcriptional and post-transcriptional gene expression^20,21^. They regulate their target messenger RNAs (mRNAs) through either their binding and inactivation and/or degradation^22^. A single miRNA may often bind up to several hundred mRNAs, while several different miRNAs can bind the same mRNA^23,24^. Muscle miRNAs or myomiRs are a large group of miRNAs enriched in skeletal and/or cardiac muscle. Although many myomiRs are exclusive for muscle tissue, some can also be found in other tissues. MyomiRs are normally involved in processes such as myogenesis and muscle homeostasis but can become differentially expressed both in general atrophy (due to immobility or caloric restriction) and muscle disorders (reviewed in Kovanda *et al*.^25^). So far over 200 myomiRs have been shown to be deregulated in different pathological conditions, such as Duchenne muscular dystrophy (DMD), Becker muscular dystrophy, facioscapulohumeral muscular dystrophy, limb-girdle muscular dystrophies, Miyoshi myopathy, nemaline myopathy, polymyositis, dermatomyositis, inclusion body myositis, etc.^26–28^. At the same time, several myomiRs have also shown potential as targets for therapeutic intervention in various models of muscular atrophy^29–33^.

In ALS, information on global miRNA expression is lacking, and the expression of only 7 miRNAs, involved in the regulation of HDAC4, has recently been examined^34^ in the muscle tissue of ALS patients. MiR-206 and miR-133b have been reported to be increased on disease onset in a SOD-1-G93A ALS mouse model^35^, with miR-206 KO mice showing delayed reinnervation, an increase in HDAC4, and faster disease progression compared to SOD-1-G93A controls. However, only miR-155, which is increased in the spinal cord of ALS patients, has so far been used as a non-muscle therapeutic target in ALS, its inhibition extending the survival SOD-1-G93A mice^36^, despite promising results with myomiR targeting in other muscle disorder models such as Duchenne muscular dystrophy and spinal and bulbar muscular atrophy^37,38^.

Despite the relatively high evolutionary conservation of miRNAs, the molecular differences between humans and existing animal and cellular models of ALS^39^ mean the information on global miRNA expression in ALS patients is crucial for any future therapy design.

The aim of our study was to determine the global differential expression of miRNAs and other small RNAs in the muscle tissue of ALS patients using small RNA Seq, in order to suggest novel disease mechanisms and identify most likely candidates for novel directions in therapy development.

## Results

### Patients and controls

We obtained muscle biopsies from 12 patients with ALS and 11 control subjects, however, due to low RNA integrity number (RIN) one patient sample was excluded from the analysis. The average age of patients vs. controls was 62.2 (±11.1) vs. 61.1 (±12.3) years, and females constituted 45.5% (5/11) and 54.5% (6/11) of patients and controls included in the study, respectively. The mean age of patients at disease onset was 58.0 (±10.9) years. 72.7% (8/11) of patients had spinal and 27.3% (3/11) had bulbar disease onset. 54.5% (6/11) patients had definite, 27.3% (3/11) had a probable, and 18.2% (2/11) had a possible ALS diagnosis according to revised El Escorial criteria^40^. Genetic analyses were previously performed for 54.5% (6/11) patients, with no known ALS related mutations discovered^41^. 18.2% (2/11) patients had a family history of the disease. Clinically, all patients had both lower and upper motor neuron involvement. The ALS FRS score of patients ranged from 18 to 43 (average 28.3 (±6.9)), however only patients who could walk were included in the study as we assumed that the severity of muscle atrophy in immobile patients would not allow for adequate comparison of muscle tissue. Therefore, all patients had ALS FRS walking scores above 1, and all but 2 patients could still use stairs to some extent. The biopsy was performed on the less affected leg in all cases. Detailed characteristics of patients and controls are shown in Table 1.

**Table 1:** Characteristics of patients (ALS) and controls (K).

Study ID	Gen-der	ALS diagnosis (revised El Escorial)	Age at disease onset	Age at muscle biopsy	ALS muta-tions	Bulbar/ Spinal onset	Stag-ing	Lower/Upper motor neuron	Famil-ial ALS	ALS FRSr	Spe-ech	Saliva-ting	Swallowing	Wri-ting	Use of utensils	Gastro-stomy	Dressing/personal hygiene	Turn-ing in bed	Wal-king	Walk-ing up stairs	Leg use = waking + steps	Dys-pnea	Orth-opnea	Breathing insuffi-ciency	Quad-riceps strength
ALS-01	F	probable	63	67	no	S	3	L/U	no	32	4	4	4	3	0	—	0	3	2	1	3	3	4	4	5
ALS-02	F	definite	50	51	nd	S	4	L/U	yes	34	4	3	3	4	4	—	1	0	3	0	3	4	4	4	5
ALS-03	F	definite	65	68	no	S	4	L/U	no	22	3	1	3	3	0	—	0	0	1	0	1	4	3	4	4
ALS-04	M	probable	77	80	nd	B	4	L/U	no	18	0	0	1	1	1	—	0	0	3	1	4	4	4	3	5
ALS-05	F	possible	59	64	nd	S	1	L*	no	43	4	4	4	4	4	—	4	4	2	1	3	4	4	4	nd
ALS-06	F	definite	70	71	nd	B	4	L/U	no	25	2	1	1	2	—	yes	1	3	2	1	3	3	4	3	5
ALS-07	M	possible	45	50	no	S	2	L/U	yes	35	4	4	4	1	1	—	0	1	4	4	8	4	4	4	5
ALS-08	M	possible	52	58	no	S	2	L/U	no	35	4	4	4	2	2	—	2	2	3	1	4	3	4	4	nd
ALS-09	M	definite	40	45	no	S	3	L/U	no	23	2	2	3	0	0	—	0	0	3	1	4	4	4	4	5
ALS-10	M	probable	61	73	nd	S	2	L/U	no	39	4	4	4	3	3	—	3	3	2	1	3	4	4	4	5
ALS-11	M	definite	59	66	no	S	4	L/U	no	23	3	1	1	0	0	—	0	2	4	4	8	3	2	2	5
ALS-12	F	definite	54	55	nd	B	3	L/U	no	25	2	0	2	2	0	—	1	3	3	1	4	3	4	4	5
K-01	F			76																					
K-02	M			69																					
K-03	F			58																					
K-04	M			72																					
K-05	F			60																					
K-06	F			43																					
K-07	F			70																					
K-08	M			57																					
K-09	F			45																					
K-10	M			46																					
K-11	M			75																					

Legend: ALS = patient group, K = control group, nd = no data, S = spinal, B = bulbar, L = lower motor neuron, U = upper motor neuron. *Sample ALS-05 was excluded from furter analyses due to low RIN.

### Next generation sequencing

**Figure 1:**
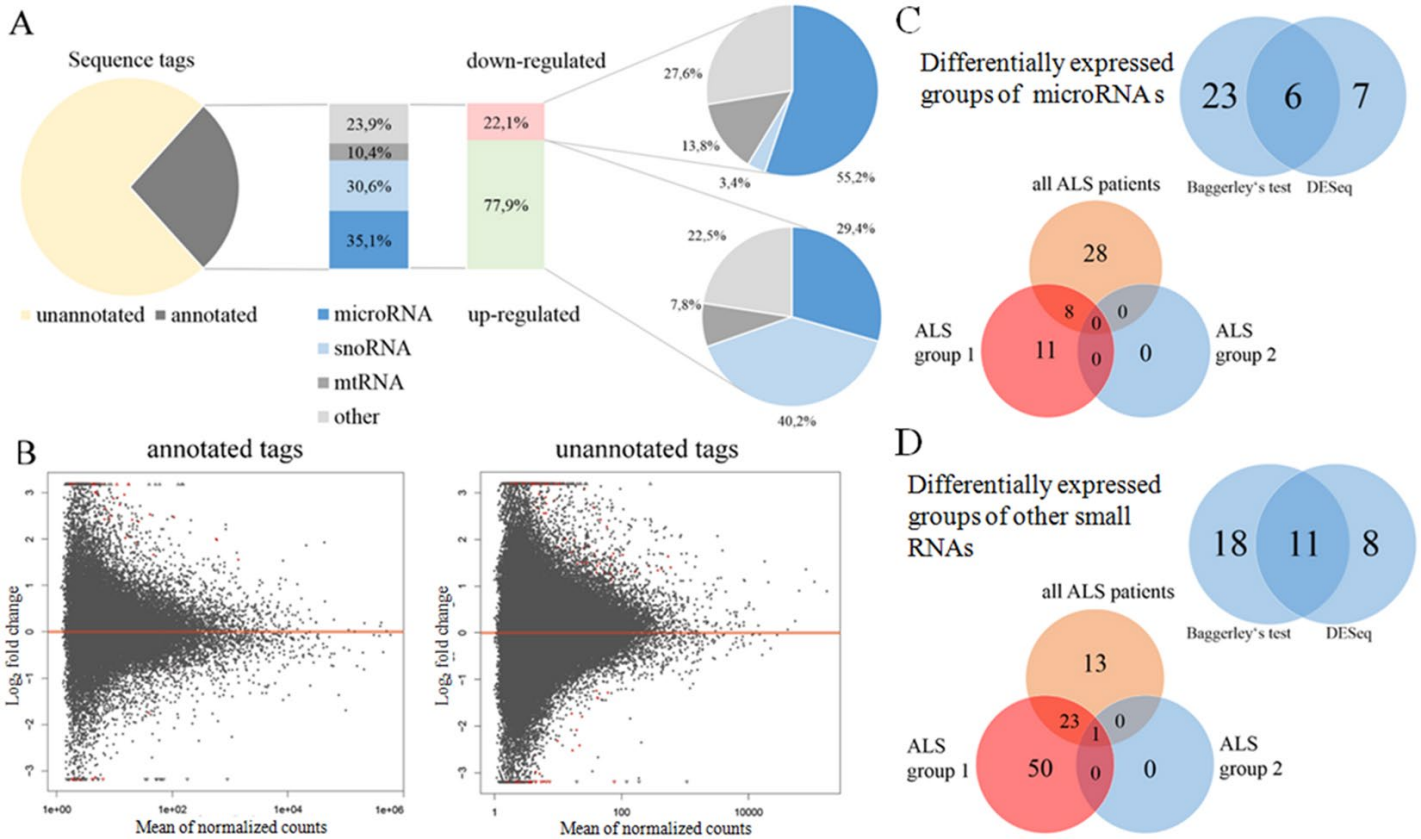
(**A**) Distribution of sequence tag annotations and differentially expressed small RNA species in ALS patients. (**B**) Plots of mean normalized counts against log2 fold change. Dots represent individual tags. Significantly differentially expressed tags are shown in red. Significant miRNA (**C**) and other small RNAs (**D**) identified by both differential analyses and identified in separate patient groups.

Based on the quality control (QC) of sample isolation prior to library preparation, one patient sample (ALS 5) was excluded from further analyses based on its low RIN. 75 base single strand small RNA next-generation sequencing of 22 samples was performed on the NextSeq 500 instrument using the high output flow cell format. One human brain total RNA sample was analyzed in parallel as a positive control. Raw data have been deposited to NCBI’s Gene Expression Omnibus^42,43^ and are accessible through GEO Series accession number GSE100188. Trimming and sequence QC was performed commercially by IMGM Laboratories GmbH using CLC Genomics Workbench 8.5.1 (CLC bio). The tags were then annotated using small RNA databases human miRBase 21 and Ensemble Non-coding RNA (GRCh38ncrna). In total between 22.1% and 30.8% reads could be annotated in this way, using both databases (Fig. 1, Supplementary Table S1).

### Differential expression

Both annotated and unannotated small RNA tags were analyzed for significantly differentially expressed small RNAs in two separate CLC Genomics Workbench experiments. The p-value was calculated using the Baggerly’s test^44^ and the p-value was FDR-corrected by using the Benjamini and Hochberg method^45^. Only tags with at least 5 read counts were included in the analysis. Significant results include only those differentially expressed small RNAs with an FDR corrected p-value ≤0.05 and fold-change FC ≥2.0. The summary overview of the changed small RNAs is given by Fig. 1.

Of the unannotated tags, 117 were significantly (FDR corrected p-value ≤0.05) up-regulated (FC ≥2.0), while 641 were significantly down-regulated (FC ≤2.0, see Supplementary Table S2 and S3, respectively). Many of the unannotated tags could be assigned to a specific genomic location or several genomic locations using BLAST (https://blast.ncbi.nlm.nih.gov/Blast.cgi). The analysis of these sequences is not yet part of established bioinformatic pipelines and is made difficult either because of their mapping to many or poorly characterized genomic locations. Although these tags may represent potential novel miRNAs or other small RNAs that could be involved in the pathogenesis of ALS, they will be analyzed in depth as part of a separate study.

**Figure 2:**
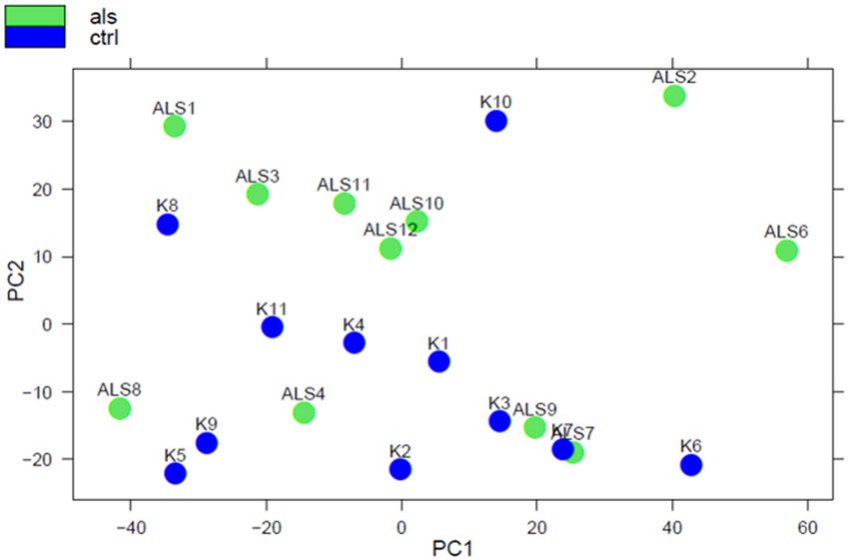
PCA plot of all annotated unique sequences, first two components are shown. ALS and control samples are clustered together, with the exception of control sample K10 and ALS samples ALS4, ALS7, ALS8, and ALS9.

**Figure 3:**
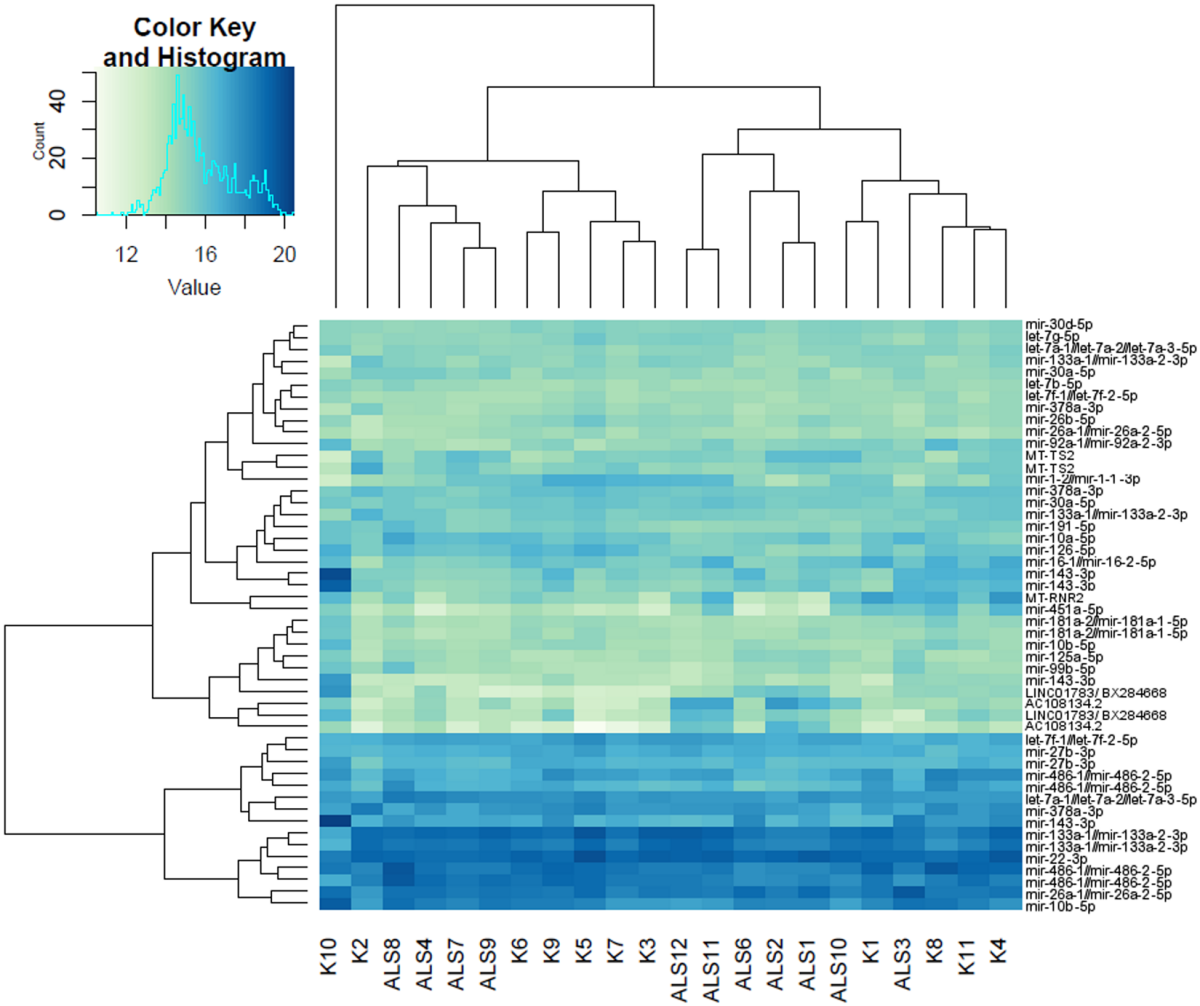
Clustering of ALS and control samples according to the 50 most highly expressed annotated small RNA sequences.

Data quality assessment was subsequently performed with sample clustering and visualization using DESeq^46^ (Figs 1, 2 and 3). MA plots of all annotated and unannotated tags are shown in Fig. 1B. The principal component analysis plot (PCA) showed four ALS samples and one control sample did not cluster with their respective groups. Based on the PCA (Fig. 2) and sample clustering (Fig. 3), that indicated a high presence of miRNAs (such as miR-143) associated with fat tissue, the control sample in question (K10) was excluded from the further analysis using DESeq. The subsequent check of the control’s weight showed a high BMI, suggesting a higher intramuscular fat content, which supports this result. Results of DESeq comparisons of all ALS patients against controls without control K10 (Supplementary Table S4) are summarized in Table 2.

**Table 2:** Significantly (FDR corrected p-value ≤0.05, FC ≥2.0) differentially expressed small RNA groups in ALS patients vs. controls.

Small RNA group:miRNAs	Expression	All ALS vs. controls*	All ALS vs. controls**	All ALS vs. controls w/o K10**	ALS group 1 vs. controls w/o K10**	ALS group 2 vs. controls w/o K10**
**hsa-miR-100-5p**	up	√	√	√	√	
**hsa-miR-10a precursor**	up	√	√	√		
**hsa-miR-125a-5p + precursor**	up	√	√	√		
hsa-miR-125b-1/miR-125b-5p + precursor	up	√				
hsa-miR-1260a-5p	up	√				
hsa-miR-126-5p	down	√				
hsa-miR-128-2-3p	up	√				
hsa-miR-1285-1-3p, hsa-miR-1285-1 /miR-1285-2-3p	down	√				
**hsa-miR-1291 precursor**	up	√		√	√	
**hsa-miR-1303-3p**	down	√	√	√	√	
hsa-miR-132-5p	up	√				
hsa-miR-133a-1/miR-133a-2-3p + precursor	up/down	√			√	
hsa-miR-150-5p	down		√	√	√	
hsa-miR-151a-5p	up	√				
hsa-miR-191-5p	down	√				
hsa-miR-199a-1//miR-199a-2//miR-199b-3p + precursor	up				√	
hsa-miR-212-5p	up	√				
hsa-miR-214-3p	up	√				
hsa-miR-22 precursor	up	√				
hsa-miR-24-1-5p	up	√				
hsa-miR-26a-1//miR-26a-2-5p	down		√	√		
hsa-miR-26a-1/miR-26a-2-5p + precursor	up	√				
hsa-miR-27a-5p	up				√	
hsa-miR-28-3p	down	√				
hsa-miR-30d precursor	down	√				
hsa-miR-3607-3p	up				√	
hsa-miR-362	up		√	√		
hsa-miR-378a -3p + miR-378a//miR-378d-2//miR-378c//miR-378d-1//miR-378e precursor	down				√	
hsa-miR-378c precursor	down	√				
hsa-miR-378d-3p	down				√	
hsa-miR-424-5p	up				√	
hsa-miR-450a-1//miR-450a-2-5p	up				√	
hsa-miR-450b-5p	up				√	
hsa-miR-4662a-5p	up	√				
hsa-miR-486-1//miR-486-2-5p	down		√	√	√	
hsa-miR-494-3p	down	√				
hsa-miR-500a-3p	up		√	√		
hsa-miR-501-3p	up	√			√	
hsa-miR-502-3p	up		√	√	√	
hsa-miR-542-3p	up				√	
hsa-miR-542-5p	up		√	√		
hsa-miR-5699-5p	down	√				
hsa-miR-584-5p	down	√				
hsa-miR-625-3p	up	√				
hsa-miR-660-5p	up				√	
hsa-miR-855-3p	down				√	
**hsa-miR-99a-5p + precursor**	up	√	√	√		
**snoRNAs**	**Expression**	All ALS vs. controls*	All ALS vs. controls**	All ALS vs. controls w/o K10**	ALS group 1 vs. controls w/o K10**	ALS group 2 vs. controls w/o K10**
SNORD100-201	up				√	
SNORD10-201	up			√	√	
SNORD102–201	up				√	
SNORD104–201	up		√	√	√	
SNORD105B-201	up				√	
SNORD110–201	up				√	
**SNORD115 group**	up	√	√	√	√	
**SNORD116 group**	up	√	√	√	√	√
SNORD12–201 group (hsa-miR-1259)	up				√	
SNORD12C-201	up				√	
SNORD13–201	up				√	
SNORD14C-201	up				√	
SNORD14D-201	up				√	
SNORD18A-201	up				√	
SNORD20-201	up	√				
SNORD22 group	down				√	
SNORD24 group	up				√	
SNORD26 group	up				√	
SNORD27 group	up				√	
SNORD32A-201	up				√	
SNORD33–201	up	√				
SNORD35B-201	up	√				
SNORD36B group	up	√				
SNORD43 group	up				√	
SNORD45 group	up	√			√	
SNORD46 group	up				√	
**SNORD48 group – RNU48**	up	√	√	√	√	
SNORD52 group	up				√	
SNORD5–201	up				√	
SNORD60–201	up	√			√	
SNORD61–201	up				√	
SNORD63–201	up				√	
SNORD64 group	up	√				
SNORD68–201	up				√	
SNORD69–201	up				√	
SNORD8–201	up				√	
**SNORD84 group**	up	√	√	√	√	
SNORD93	up	√				
**SNORD95–201**	up	√	√	√	√	
**SNORD97–201**	up	√	√	√	√	
SNORD99–201	up				√	
**Mitochondrial origin**	**Expression**	All ALS vs. controls*	All ALS vs. controls**	All ALS vs. controls w/o K10**	ALS group 1 vs. controls w/o K10**	ALS group 2 vs. controls w/o K10**
**MT-RNR2–201**	up	√	√	√	√	
MT-TE-201	down				√	
MT-TF-201	up	√			√	
MT-TH-201	down			√		
**MT-TL1–201**	up	√		√	√	
MT-TL2-201	up/down	√			√	
MT-TN	down				√	
MT-TP-201	up			√	√	
MT-TQ	up				√	
MT-TS2–201	down				√	
MT-TV-201	up				√	
**MT-TY-201**	up/down	√		√		
MT-TM-201	up	√				
MT-TC-21	up	√				
**Other ncRNAs**	**Expression**	All ALS vs. controls*	All ALS vs. controls**	All ALS vs. controls w/o K10**	ALS group 1 vs. controls w/o K10**	ALS group 2 vs. controls w/o K10**
AC006041.1–001 lincRNA	up				√	
AC074212.5-002 retained intron	down				√	
AC084082.3-001 lincRNA	up				√	
AP000233.2-002 lincRNA	up				√	
CTD-2562J15.4 known antisense RNA	up				√	
**CTD-2651B20.7-001 CTD-2651B20.6-001**	up	√	√	√	√	
GAS5-006 (growth arrest-specific 5) retained intron	up				√	
GAS5-007 (growth arrest-specific 5) retained intron	up				√	
GAS5-014 (growth arrest-specific 5) lincRNA	up				√	
GAS5-015 (growth arrest-specific 5) lincRNA	up				√	
GAS5-022 (growth arrest-specific 5) retained intron	up				√	
LIMD1-AS1-002 (LIMD1 antisense RNA 1)	up				√	
LINC00293	up	√				
LINC00324	up		√	√	√	
LINC01470-006	up				√	
LINC01783-001	up				√	
Retired novel miRNA ENST00000616457.1//ENST00000612700.1// ENST00000611802.1//ENST00000611393.1//ENST00000612047.1//ENST00000617883.1	down				√	
Retired miRNA ENST00000614470.1//ENST00000611934.1//ENST00000488123.2//ENST00000617236.1	down				√	
RNA5-8S5	down	√			√	
RNA5-8SP6-201 (RNA, 58S pseudogene 6)	up				√	
RNVU1-7-201RNA, variant U1 small nuclear 7	up				√	
RP11-3B12.3-001	up		√	√	√	
RP11-395B7.2	down	√				
**RP11-473M20.16-001**	up	√	√	√	√	
RP11-1260E13.4 group	down	√				
RP4-561L24.3-001	up		√	√	√	
RP4-671O14.6-001 known sense overlapping	up				√	
SNHG1-003//SNHG1-024//SNHG1-013	up		√	√	√	
snoU18.1-201 space novel snoRNA	up				√	
U1 spliceosomal RNA	up				√	
XXbac-BCX254L4.4-001//RP5-1186N24.3-001	down	√			√	
ZNF436-AS1-201	down	√				

ALS group 1 samples include ALS 1, 2, 3, 6, 10, 11, 12, while ALS group 2 samples include samples ALS 4, 7, 8, 9. *Baggerly’s test, **DESeq analysis.

Among the annotated tags, the CLC Genomic Workbench experiment identified a total of 134 tags that differed significantly between the ALS and control groups. Within the 115 up-regulated annotated tags, 19 groups of miRNAs and 24 other ncRNAs were represented (Supplementary Table S5), while within the 19 down-regulated annotated tags, 10 groups of miRNAs and 5 ncRNAs were represented (Supplementary Table S6), respectively. The DESeq calculation re-identified 6 of these differentially expressed miRNAs and 11 of the differentially expressed other small RNAs with FDR statistical significance, as well as identified additional 7 miRNAs and 8 other small RNAs, respectively (Fig. 1C,D).

The four ALS samples (ALS 4, ALS 7, ALS 8, and ALS 9) that were nested within the control group in the PCA analysis all had high leg-use scores (walking and using steps) and only one of the patients had a definite ALS diagnosis (Table 1). This particular patient happened to be the youngest of our patients at 45 years of age. These factors, combined with our choice of the less affected leg, may explain these results. This is also in line with the hypothesis that active muscle regeneration attempts precede the development of noticeable clinical atrophy in case of ALS^1,34^, and thus in our case may mask some disease-associated changes.

Therefore, we also separated the ALS patients into two groups, group 1 (patients ALS 1, ALS 2, ALS 3, ALS 6, ALS 10, ALS 11, ALS 12), and group 2 (patients ALS 4, ALS 7, ALS 8, and ALS 9) and compare them to controls with the aim of identifying any group-specific molecular markers that could be associated with disease severity.

Comparisons of ALS group 1 with controls excluding K10 (Supplementary Table S7), indeed resulted in the identification of 11 additional microRNA groups as well as 50 other small ncRNAs, among them 2 retired novel miRNAs, 26 snoRNAs, and 5 mitochondrial RNAs **(**Table 2 and Fig. 1C,D). However, the results of DESeq comparisons of ALS group 2 patients with controls excluding K10 (Supplementary Table S8) did not show any significantly changed miRNAs and only showed one significantly changed ncRNA, which was snoRNA-116 (Table 2, Fig. 1C,D). This may be interesting in that it may represent an early marker of disease and will be further discussed below (See other differentially expressed small RNAs.).

### Target, KEGG, and GO analyses of differentially expressed miRNAs

*In silico* Tarbase analysis of miRNA targets based on the comparison of all patients against all controls showed over 14,000 genes to be targeted, some of which by more than one of the differentially expressed miRNAs. Of these targets, approx. 40 have already been implicated in neuronal ALS pathology or other disorders involving muscle wasting, while several hundred others are suggested to be involved in muscle contraction, muscle organ development, skeletal muscle cell differentiation, muscle morphogenesis etc. (Supplementary Table S9).

**Figure 4:**
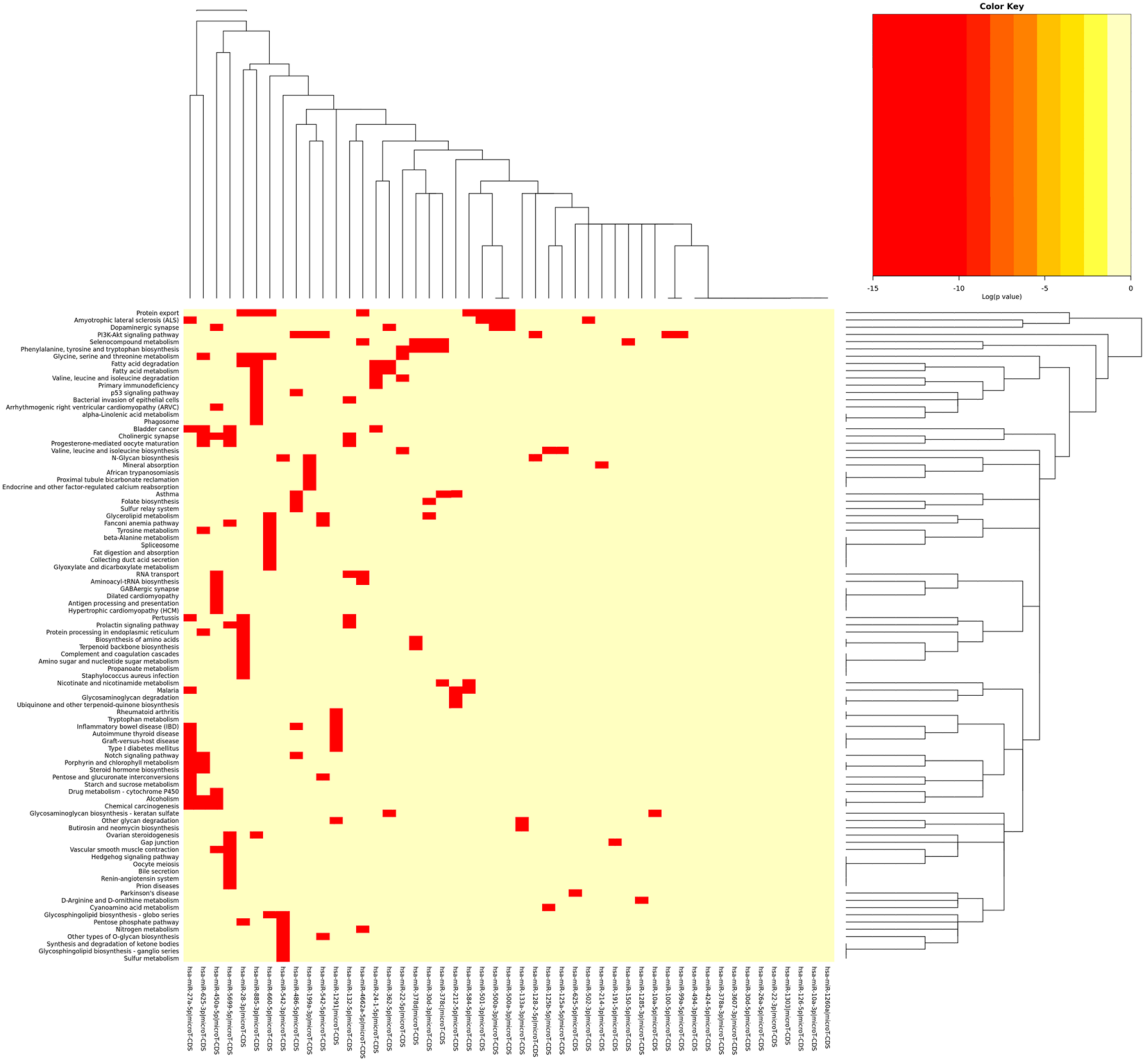
Heatmap of KEGG union significance clusters based on miRNA species identified by both Baggerly’s test and DESeq.

KEGG pathways **(**Fig. 4**)** and GO genes union analyses were performed on targets of all differentially expressed miRNAs identified by both the Baggerly’s test as well as the various DESeq group analyses (Supplementary Tables S10 and S11). Of note is that the top hits among the KEGG pathways (Fig. 4) include signaling pathways regulating pluripotency of stem cells, ubiquitin-mediated proteolysis, axon guidance, regulation of actin cytoskeleton, and TGF-beta signaling pathway which could be expected to be affected in a tissue undergoing both degenerative/apoptotic and regenerative processes. Additionally, both fatty acid biosynthesis and metabolism have been previously implicated in ALS and may present targets for therapy development^47^. Although cancer pathways, such as proteoglycans in cancer and glioma are also among the top hits, this is most probably coincidental due to an overabundance of miRNAs studies in cancer and possible muscle atrophy pathway overlap between ALS and cancer-associated cachexia.

GO molecular function categories included various parts of RNA metabolism, such as RNA binding, and nucleic acid and protein binding transcription factor activity, which are known to be affected in ALS. GO cellular compartment categories also included established ALS associated terms, such as protein complex. Interestingly, nucleoplasm – the role of which is currently under intense investigation^48,49^, as well as platelet alpha-granule lumen (that contains insulin-like growth factor 1), which has been linked to Alzheimer’s disease^50^, were also among the top GO compartment hits. GO biological processes included over a hundred terms, among them, expected apoptotic signaling pathways, but also neurotrophin TRK receptor signaling pathway and muscle cell differentiation, supporting previously observed findings that muscle tissue is actively making attempts at regeneration during ALS progression^51^. Additionally, cellular lipid metabolic process, insulin receptor signaling pathway, synaptic transmission and axon guidance were also among the biological process GO hits.

### Other differentially expressed small RNAs

Our analyses identified over 40 groups of small nucleolar RNAs (snoRNAs) to be differentially expressed, of which all but one were upregulated in ALS patients (Table 2). All belonged to the C/D box snoRNA family (SNORD) which is associated with methylation of ribosomal RNAs and may, therefore, reflect the increased ribosomal turnover as part of the increased protein metabolism during compensatory efforts^52^.

Small RNAs of mitochondrial origin were also significantly differentially expressed, which is in line with aberrant mitochondrial function in ALS. Tags mapping to the MT-RNR2 (mitochondrial 16 S RNA) were the only ones to be significantly upregulated by both the Baggerly and the DESeq analyses.

Additional differentially expressed ncRNAs include spliced transcripts SNHG1-003//SNHG1-024//SNHG1-013, involved in neural-stem-cell differentiation^53^, and GAS5 (growth arrest-specific 5) - small nucleolar RNA host gene, that can serve as a decoy for the glucocorticoid and related receptors and promotes apoptosis^54^.

## Discussion

In our analysis of the global differential expression of small RNAs, we identified 758 un-annotated and 134 annotated tags (including miRNAs, snoRNAs, and mtRNAs) to be differentially expressed in the muscle tissue of ALS patients. This previously unknown diversity at the level of regulatory RNA molecules may point us toward finding novel disease mechanisms as well as the most likely candidates for novel directions in therapy development.

The KEGG and GO analyses of the differentially expressed miRNAs suggest their likely involvement in both degenerative/apoptotic processes, as well their involvement in muscle regeneration attempts. Furthermore, several of the differentially expressed miRNAs identified in the muscle tissue of ALS patients have previously been linked with ALS.

Our results show partial overlap with neuronal studies of associations between miRNAs and ALS. In neuronal models, TDP-43 mutations have been shown to cause differential expression of miR-132, miR-143 and miR-558, and TDP-43 deficiency was shown to impair neurite outgrowth in Neuro2a cells, which could then be rescued by miR-132 overexpression^55^. Similarly, FUS has been shown to promote the biogenesis miR-9, miR-132 and miR-134^56^, which are miRNAs with a known function in neuronal development and synaptic plasticity^57–62^. Additionally, miR-9 down-regulation was observed in induced pluripotent stem cell derived neurons from an ALS patient with a TDP-43 mutation^63^, whereas miR-155, which is increased in the spinal cord of ALS patients, was suggested as a therapeutic target and its inhibition extended surivival in SOD-1-G93A mice^36^. Recently, miR-218 has also been suggested to affect motor-neuron loss in a rat hSOD1 WT ALS model rats^64^.

Of the miRNAs with proposed CNS involvement in ALS, miR-132 and miR-125b, were up-regulated in our study. While miR-132 is directly affected by mutations in both TDP-43 and FUS in neuronal models of ALS^55,56^, miR-125b has been connected to neuro-inflammation through NF-kB activation in a SOD-1-G93A microglia culture model^65^. Whether miR-125b may act in a similar way in muscle and whether its origin may be the motor neurons or neuro-muscular junctions within the muscle tissue, remains to be determined.

A classical myomiR, miR-133a is predicted to target TUBA4A, which was recently shown to be involved in ALS^66,67^, and has also been shown to change during myogenesis^68^, in response to physical activity^69,70^, aging^71^ as well as during neuromuscular regeneration and reinnervation in a rat denervation model^72^, and has recently been shown to be increased in early-stage and slow-progressing ALS patients^34^. Interestingly, our comparison of all patients against the controls showed a significant increase, while the comparison of ALS group 1 patients (higher disease severity) against controls showed a significant decrease in miR-133a, suggesting it may undergo change during disease progression.

Furthermore, miR-133 and miR-30d (that was down-regulated in all patients vs. controls) are repressed by insulin in skeletal muscle tissue^73^ and may be involved in the observed protective role of diabetes against the development of ALS^74^. Of the miRNAs that have already been therapeutically targeted in mice and rats^30,36^, only miR-133a was identified as being differentially expressed in our study and therefore likely represents a good target for human therapy development.

In contrast, we did not find changes in the expression of miR-1 or miR-206, despite them being among the most highly expressed miRNAs in muscle tissue in general, and despite miR-206 being recently identified as a potential biomarker in serum from ALS patients^75^, as well as increased in muscles of four early-stage ALS patients^34^. This likely reflects the differences in our study populations, i.e., serum of ALS patients with varying scores of leg-use in the Waller study, and the muscle tissue of ALS patients with above-average leg-use in our study. As miR-206 is highly expressed in human muscle, we postulate that the observed time-dependent differences in miR-206 observed in the Waller study as well as our observations, indicate that miR-206 may bear direct correlation with the deterioration of the muscle tissue, rather than this miRNA being directly involved in ALS pathogenesis. The observed difference with the study by Di Pietro *et al*., is expected, given the different patient cohorts, as they have observed a difference in miR-206 expression only in the four patients with early-stage disease (less than 10 months after onset), whereas our study included only two such patients (one year after onset), whereas both studies agreed and showed no difference in patients with longer progression.

Similarly, miR-133a and miR-26a were also the only ones, of the differentially expressed miRNAs previously observed in muscle tissue of ALS patients using qPCR methods^34,51,76^, that could be confirmed by our study. Indeed, in muscle tissue of ALS patients, miR-29 has been previously observed to increase together with miR-23a, miR-29b, miR-206 and miR-455^76^. Recently, however, the expression of some of these myomiRs (miR-1, miR-23a, miR-26a, miR-27b, miR-29b, miR-133a, miR-206, and miR-455) has been re-examined by using qPCR methods in muscle samples of ALS patients and controls^51^, and miR-1, miR-26a, miR-133a, and miR-455 were shown to be significantly decreased in patients. The reason for this discordance is likely methodological, as there are no reliable internal control standards for miRNA qPCR yet^77–79^. Choosing controls for qPCR remains a tremendous challenge because, during atrophy, skeletal muscle is subject to global morphological changes affecting fiber type and overall muscle, vascular and adipose cell content, which in turn affects housekeeping genes^51,80–82^. Furthermore, as will be discussed further, in our study we have detected a significant up-regulation of SNORD48, or RNU48, which is a common qPCR control molecule.

Five of our differentially expressed miRNAs have so far also been shown in normal muscle tissue processes. MiR-24, miR-26, miR-27, miR-214, and miR-502 were up-regulated, both in cellular models of differentiation of satellite cells into myoblasts and myotubules^83–89^, as well as in our study. This observation strongly suggests the myomiRs are actively involved in muscle differentiation and likely play a part in compensatory attempts during ALS progression. While miR-125b was found to be down-regulated in the mentioned cellular models of muscle differentiation^83–89^, in our study it was found to be up-regulated, likely reflecting previously discussed neuro-inflammatory mechanisms relating to ALS pathogenesis^65^.

Interestingly, apart from our study, a decrease of miR-468 has so far been observed after acute or chronic exercise^90^. Although counterintuitive, this may be explained by fasciculations that precede atrophy in ALS. Indeed, rather than being inactive and senescent, muscle tissue in ALS is in effect overly active in parallel to atrophy development^1^.

Furthermore, miR-1260a, miR-214, and miR-501 are strongly (miTG score of >0,9) predicted to have binding sites for controlling TDP-43, a protein central to ALS pathogenesis. Similarly, miR-30d is predicted to target C9ORF72, a gene whose intronic expansion mutation is the most common genetic cause of ALS. MiR-30d can additionally target CARF, TRPM7, and CHMP3B, all of which were shown to be involved in ALS to various extents^91^, and TRPM7 and CARF are also predicted to be targeted by miR-22 and miR-26a, respectively. Furthermore, miR-378c may target hnRNPA1, another protein central to ALS pathology^9^. Finally, miR-1291 is predicted to target ATXN2 and DCTN1, while miR-10a is predicted to target ALS2 protein.

Of the differentially expressed snoRNAs, unexpectedly SNORD115, SNORD116, SNORD48, SNORD84, SNORD95, and SNORD97 were significantly upregulated in both the Baggerly and DESeq analyses, as well as in the ALS group 1 comparisons. Interestingly, SNORD64, SNORD115, and particularly SNORD116 are absent in Prader-Willi syndrome^92,93^, which is characterized by severe hypotonia at birth and later development of obsessive eating, morbid obesity and type-2 diabetes^94^. Additionally, SNORD115 may also target serotonin receptor 5HT-2C mRNA^95^. In our study all three were shown to be up-regulated, providing a possible molecular link between the observed inverse relationship between ALS, fatty acid metabolism, and diabetes^47,74^. Unexpectedly, SNORD116 was the only small RNA significantly differentially expressed when comparing the ALS group 2 patients to controls, indicating it may be one of the first observable changes in the muscle tissue of ALS patients during disease progression.

SNORD48 also known as RNU48, was shown to be significantly upregulated in ALS patients, adding to mounting evidence that this commonly used qPCR internal control should be used with care^96,97^. While the role of SNORD84 and SNORD97 are unknown, SNORD95 is expressed from the intron of the RACK1 (GNB2L1) gene, which is involved in translational repression and ribosomal quality control^98,99^. Moreover, SNORD20 originates from an intron of the human nucleolin gene, defects in which have been linked with C9ORF72 repeat related ALS^100^.

Of note, MT-RNR2 encodes for humanin peptide, which is associated with apoptosis and insulin sensitivity and is thought to be protective in Alzheimer’s and Huntington’s disease^101–103^. Furthermore, it was found to be increased in endurance exercise, again suggesting a link to fasciculations in ALS^104^.

## Caveats

The technical challenges faced in our study include a relatively low number of samples, lack of stable normalizers for qPCR validation^105^–^107^, and difficulties with functional validation of miRNA targets.

The number of samples in our study was limited by inclusion criteria and ethical considerations. The ability to walk was chosen as our main inclusion criteria because the hallmark of ALS is rapidly progressive loss of muscle tissue. In immobile patients, the procedure would be unethical, and over three-quarters of the interested patients were excluded due to this issue. Despite their low number the collected samples nevertheless represent a valuable resource as they were collected from functional muscles of ALS patients, and we were therefore in a unique position to identify processes taking place before functional muscle loss.

A traditional approach in expression studies has been to validate global expression results using qPCR. For this stable normalizers are needed, but identifying such molecules is often challenging, and needs to be identified on a case-by-case basis^105–107^. Despite attempting to identify appropriate small RNAs for qPCR validation, we have not found such molecules, either through the NormFinder^108^ software nor by in-house methods. The most likely explanation is that muscle atrophy, regardless of its cause, is a global process also affecting the expression of housekeepers or normalizers. An additional issue is that it is not clear how the numerous identified sub-, super-, and precursor variants, would, if at all, be detected by qPCR assays, which specifically target mature miRNAs.

Finally, under ideal conditions, functional validation of miRNA’s target proteins would be performed, as the expression on the mRNA level does not directly correlate with miRNA expression. Unfortunately, small muscle biopsy size, the large number of changing small RNA molecules and last but not least limited availability of adequate commercial antibodies for target human proteins prevented us from achieving this goal.

Therefore, due to the mentioned caveats and technical limitations, we would like to emphasize that some of our conclusions remain speculative, however, they are for the most part consistent with current knowledge and we hope our findings will form the basis for further research in this direction.

## Conclusions

Our study represents the first, comprehensive differential analysis of small RNA expression in muscle tissue of ALS patients. We have identified many differentially expressed miRNAs, some of which have been previously suggested to be associated with known or postulated pathways in ALS, muscle differentiation, and reinnervation, and we comment on the similarities and differences with differential expression of miRNAs in ALS in blood, shown so far, hopefully highlighting molecules promising as biomarkers or targets in therapy development. We furthermore show that differential expression of small RNAs in ALS includes molecules such as RNU48, considered as housekeepers, as well as mtRNAs and snoRNAs involved in other diseases.

## Materials and Methods

### Ethics statement

Ethical approval of the study (No. 58/11/14) was obtained from the Republic of Slovenia National Medical Ethics Committee – NMEC. Written informed consent was obtained from all patients and controls. All methods were performed in accordance with the relevant guidelines and regulations.

### Patients and controls

Slovenian patients with amyotrophic lateral sclerosis as well as their spouses or other non-blood relatives were invited to participate in the study by either the ALS team coordinator and/or treating neurologist at the Ljubljana ALS Centre, Institute of Clinical Neurophysiology, University Medical Centre Ljubljana.

The inclusion criteria for all volunteers were the ability to walk/still walk, and having no counter-indications for the biopsy procedure, such as receiving anticoagulant therapy. Muscle biopsy samples were collected from 12 patients and 11 controls, from February until August 2015. In addition to the muscle biopsies, clinical data were also collected at or just prior to the visit. Data collected included the ALS diagnosis according to the revised El Escorial score^40^, staging, age at diagnosis, ALS mutation status, bulbar/spinal onset, upper/lower motor neuron deficits, family history of ALS, ALS FRSr and quadriceps strength.

### Muscle biopsy procedure

Muscle biopsy procedures were performed on the *vastus lateralis* muscle using a standard procedure by trained physicians who routinely perform the procedure for diagnostic purposes. In case of patients, the less affected leg was chosen for the biopsy in order to minimise the possibility of obtaining connective tissue and fat rather than muscle.

In short, the site of the biopsy was disinfected and then anesthetized by injecting 2% Xylocaine. After anesthetic took hold, a small cut to the skin and fascia was made through which the biopsy sample (10–100 mg) was taken using a rongeur instrument. The sample was quickly visually inspected for fat/blood vessels, which were removed if present, and then immediately snap frozen in liquid nitrogen and then transferred to −80 °C until further analyses. The incision was closed using steri-strips bands and the biopsy site was pressure bandaged for a few hours. Instructions were given as to the home care of the biopsy site. One adverse event (hematoma) was noted in connection with the biopsy in one of the controls. No adverse events were noticed in any of the patients or other controls.

### Isolation and sequencing of small RNAs

Extraction of total RNA, library preparation, and next-generation small RNA sequencing were performed commercially by IMGM Laboratories GmbH (Martinsried, Germany) on Illumina NextSeq 500.

### Isolation and quality control

Total RNA was isolated using miRNeasy kit (Qiagen) according to the protocol of the manufacturer. The final RNA concentration and purity were determined using NanoDrop ND-1000 spectral photometer (peqlab). In order to determine RNA integrity number (RIN), samples were analyzed on the 2100 Bioanalyzer (Agilent Technologies) using the RNA 6000 Nano LabChip Kit (Agilent Technologies). Based on the quality of RIN, and RNA concentration and purity, 22 of the 23 samples were selected for small RNA library preparation for NextSeq sequencing, while one ALS sample (ALS-5) was excluded from any further analyses.

### Small RNA library preparation

Illumina® TruSeq® Small RNA Library Prep Kit (Illumina) was used to generate small RNA libraries of 22 samples according to manufacturer’s instructions with one human brain total RNA sample processed and analyzed in parallel as a positive control. An input of 800–1000 ng of RNA was used for library generation.

PCR amplification was performed on the generated single strand cDNA with index primers in order so that the samples could be multiplexed for further library analyses. PCR products were purified and size selected using gel purification (6% Novex TBE gels) and the purified cDNA constructs were concentrated by ethanol precipitation. Quality control of each library sample was performed using the High Sensitivity DNA LabChip Kit (Agilent Technologies) on the 2100 Bioanalyzer (Agilent Technologies) before and after size selection. Library quantification prior to normalization was performed using Qubit® dsDNA HS Assay kit (Invitrogen). Post-purification, all samples ranged in size between 70 and 155 bp (including adaptor sequences) and were of sufficient concentration for RNA sequencing analysis. Libraries were pooled in equimolar ratios for sequencing.

### Next generation sequencing

Two single-end 75 base sequencing runs (1x 75 bp SE) were performed with the final library on the NextSeq 500 sequencing system (Illumina) under the control of the NextSeq® Control Software (NCS) (Illumina), using a PhiX v3 control library spike-in (Illumina). Cluster densities ranged from 107–119 k/mm2 and two runs per sample were performed in order to obtain a sufficiently high yield per sample. The two 1 × 75 SE runs had 96.5% and 95.8% >Q30 bases, respectively. Real-Time Analysis 2.4.6 Software (RTA) was used to process the primary image on the NextSeq 500 instrument, while primary data analysis was performed using the bcl2fastq 2.15.0.4 software package. Sequencing run performance was imaged and evaluated using the Illumina Sequence Analysis Viewer (SAV) 1.11.0. QC Results of sequenced samples are summarised in Supplementary Table S1.

## Bioinformatic analysis

### QC, mapping and differential expression analysis

The first part of the bioinformatic data analysis was performed commercially by IMGM Laboratories GmbH using CLC Genomics Workbench 8.5.1 (CLC bio). Sequencing reads were trimmed, removing the adaptor (RTP 5′ GCCTTGGCACCCGAGAATTCCA), and only reads with the length between 15 and 55 bp were selected for further analyses. The number of unique tags (unique nucleotide sequence) was counted in each sample (Supplementary Table S1) and the tags were annotated using small RNA databases human miRBase 21 and Ensemble Non-coding RNA (GRCh38ncrna), and the ‘Annotate and Merge’ tool from CLC Genomics Workbench. Up to two mismatches, no gaps and + /−2 nucleotides were allowed to be counted up- and/or downstream from known small RNA sequences in the databases. The mapping was performed by allowing no mismatches in the first round of analysis, then allowing one mismatch for the unmapped reads in the second round, and finally allowing two mismatches for the remaining unmapped reads in the final round of mapping. Each included tag (unique nucleotide sequence) had to be detected at least five times, in order to remove possible sequencing artifacts. The analysis resulted in annotated and unannotated set of sequences, which were then compared in a pairwise manner between the reference and ALS group (please see statistical analyses).

The second part of the bioinformatic data analysis was performed at the Laboratory of Bioinformatics at the University of Ljubljana Faculty of Computer and Information Science.

Additional differential expression analyses including data quality assessment by sample clustering and visualization were performed with the DESeq software package^46^ based on the IMGM annotated and unannotated reads of ALS patients and controls, as well as on total unique tag counts. For the DESeq analysis based on the IMGM annotated and unannotated tags of ALS patients and controls, the reads were filtered prior to the analysis with only those tags detected at least five times in at least six samples included in the analysis.

### Target, GO and KEGG Pathway analysis

Target analysis was performed on-line using the DIANA tools MR-microT software^109,110^ (Updated to Ensembl v84). The list of identified target proteins was then compared against the Ensembl databases and with the help of Ensembl Biomart^111,112^ to identify proteins known to be involved in ALS, other dystrophies, muscle development, and muscle disease.

Gene ontology (GO) and Kyoto Encyclopedia of Genes and Genomes (KEGG) pathway analyses were conducted online using the DIANA-miRPath v3.0. tools^113,114^ on all 49 differentially expressed miRNAs, including their 3′ and 5′ species, identified by both the Baggerly’s test and DESeq in order to identify relevant pathways and gene ontologies. All DIANA-miRPath analyses using Tarbase v7.0 were performed with FDR correction and conservative statistics options.

## Statistical analysis

Patients’ and controls’ characteristics were analyzed by using descriptive methods (average, standard deviation (SD)).

Statistical analysis performed commercially by IMGM Laboratories GmbH consisted of a pairwise comparison of annotated sequences of ALS patients vs. controls, and unannotated sequences of ALS patients vs. controls, using the Baggerley *et al*.’s test^44^. Benjamini and Hochberg method was used to calculate the false discovery rate (FDR)^45^. Only tags with at least 5 counts were included in the analysis. In order to achieve significance, both a p-value and corrected p-value of ≤0.05 needed to be reached by the changed small RNA. Additionally, a proportion fold change cut-off of 2.0 was implemented, meaning small RNAs were considered induced if the proportions fold change (FC) values ≥2.0 and repressed if the value was ≤−2.

In order to validate the results, the count data were further analyzed using the DESeq as an R/Bioconductor package, as previously described^46^.

## Electronic supplementary material

Supplementary tables S1-S11
